# Preliminary Metabolomic Analysis: Serum Metabolomic Dynamics During Estrus Synchronization in Kazakh Mares

**DOI:** 10.3390/ani16142222

**Published:** 2026-07-17

**Authors:** Jiahao Liu, Jintao Gan, Xinkui Yao, Jianwen Wang, Wanlu Ren, Jun Meng, Yaqi Zeng

**Affiliations:** 1 College of Animal Science, Xinjiang Agricultural University, Urumqi 830052, China; ljh072412@163.com (J.L.); ganjintao2022@163.com (J.G.); yaoxinkui@xjau.edu.cn (X.Y.); dkwjw@xjau.edu.cn (J.W.); renwanlu@xjau.edu.cn (W.R.); mengjun@xjau.edu.cn (J.M.); 2 Xinjiang Key Laboratory of Equine Breeding and Exercise Physiology, Urumqi 830052, China

**Keywords:** Kazakh horse, synchronization of estrus, serum metabolomics, DAMs

## Abstract

This study aimed to analyze the differences in serum metabolomes at different stages of estrus synchronization in Kazakh horses, to characterize dynamic changes in serum metabolites during estrus synchronization, and to screen potential metabolic markers and key regulatory pathways. Four sampling stages (M1: intravaginal device insertion; M3: device removal, labeled consistently with the pre-experiment timeline; P: 24 h post-PG injection; L: tertiary follicle emergence) were selected. The research results showed that 37 significantly different metabolites (DAMs) were identified in the M1-vs.-M3 positive ion mode, with 16 metabolites significantly upregulated and 21 significantly downregulated. In the M1-vs.-M3 negative ion mode, 47 significantly different metabolites were identified, including 17 upregulated and 30 downregulated. In the M3-vs.-P positive ion mode, 69 significantly different metabolites were identified, with 53 upregulated and 16 downregulated. In the M3-vs.-P negative ion mode, 63 significantly different metabolites were identified, including 39 upregulated and 24 downregulated. In the Pvs-L positive ion mode, 65 significantly different metabolites were identified, with 16 upregulated and 49 downregulated. In the P-vs.-L negative ion mode, 61 significantly different metabolites were identified, including 13 upregulated and 48 downregulated. The significantly different metabolites mainly included lipids and lipid-like molecules, organic heterocyclic compounds, and organic acids and their derivatives. KEGG enrichment analysis showed that the significantly different metabolites in Kazakh horses at different stages were mainly enriched in nicotinic acid and nicotinamide metabolism, amino acid synthesis, and related pathways. This study revealed significant differences in the serum metabolome, and nicotinic acid and nicotinamide metabolism, by generating NAD^+^ and NADPH, simultaneously supporting energy supply, steroid hormone synthesis, and antioxidant protection, which are the basic metabolic guarantees for the development of follicles from quiescence to growth; amino acid synthesis not only provides protein raw materials for follicular cell proliferation but also participates in the regulation of the follicular microenvironment by synthesizing signaling molecules and antioxidant substances. This study enriched the research on the metabolic regulation of estrus synchronization in equine species, filled the gap in the metabolomics research of estrus synchronization in Kazakh horses, and provided an important theoretical basis and practical reference for optimizing the estrus synchronization treatment plan for Kazakh horses, adjusting the focus of feeding and management at each stage and improving the conception rate.

## Simple Summary

This study aimed to systematically characterize dynamic serum metabolomic variations across four critical time points during estrus synchronization in Kazakh mares, screen stage-specific differential serum metabolites and core metabolic pathways, and identify hub metabolic markers closely associated with follicular development and reproductive hormone fluctuations. We adopted untargeted metabolomics to detect serum metabolites at four key stages of estrus synchronization: intravaginal device insertion stage (M1), device removal stage (M3), and 24 h after PG (prostaglandin F_2_α, PGF_2_α). All PG injections were combined with hCG administration as the estrus synchronization protocol described in Materials and Methods. We identified significant differential metabolites (DAMs) across stages, dominated by lipids and lipid-like molecules, organic acids and derivatives, and organic heterocyclic compounds. Kyoto Encyclopedia of Genes and Genomes (KEGG) enrichment revealed that nicotinate and nicotinamide metabolism, amino acid biosynthesis, glycerophospholipid metabolism, and the TCA cycle were the most enriched pathways. Nicotinate and nicotinamide metabolism is associated with pathways related to energy metabolism, steroidogenesis, and redox balance via the generation of nicotinamide adenine dinucleotide (NAD^+^) and nicotinamide adenine dinucleotide phosphate (NADPH), while amino acid biosynthesis provides substrates for follicular cell proliferation and microenvironment regulation. Temporal clustering identified hub metabolites including N-hexanoylsphingosine, palmitoyl sphingomyelin, L-leucyl-L-proline, uric acid, and glycerophosphocholine, which were closely correlated with reproductive hormone levels. These findings reveal the dynamic metabolic regulatory patterns during estrus synchronization in Kazakh horses fill the gap in metabolomic research on estrus synchronization in this breed, and may provide insights for refining estrus synchronization strategies and reproductive management in Kazakh horses.

## 1. Introduction

The Kazakh horse, as an excellent local horse breed in China, has strong adaptability and can tolerate roughage. It is an important part of the livestock industry in regions such as Xinjiang. Its reproductive efficiency directly affects the development of the industry [1,2]. Currently, breeding programs for Kazakh horses face challenges including asynchronous estrus and suboptimal conception rates, which restrict the development of large-scale farming [3]. The estrus cycle of mares is generally about 22 days, and the duration of estrus is mostly 5 to 7 days. The reproductive season is influenced by multiple factors such as light, environment, and body condition [4]. External hormones such as progesterone devices, prostaglandin analogues, and HCG have been used to regulate estrus and ovulation in mares, which is of practical significance for improving reproductive management efficiency [5,6]. Therefore, it helps to more systematically understand the continuous changes in mares from hormone inhibition and luteal regression to rapid follicular development, starting from potential metabolite markers and combined with pathway enrichment analysis, which is more conducive to analyzing the transition from hormone inhibition and luteal regression to dominant follicular development during synchronous estrus in mares [7,8,9].

In recent years, metabolomics, as an important technique for studying the overall changes in endogenous small molecule metabolites in the body, has been widely applied in animal nutrition, growth and development, and reproductive physiology research. For instance, the metabolic composition of follicular fluid in mares before ovulation is closely related to the maturation of oocytes, suggesting that small molecule metabolites in the follicular microenvironment can directly reflect the developmental ability of oocytes [10,11,12]. Further, the analysis of saliva and plasma metabolomes of mares during seasonal anestrus, estrous cycle, and early pregnancy revealed that metabolites such as alanine and creatine could serve as candidate markers reflecting the initiation of the ovarian cycle and the approach of ovulation, indicating that molecules related to energy metabolism and amino acid turnover have high sensitivity for determining the estrous stage [13]. Catandi et al. [14] observed significant changes in the abundance of metabolites such as glutamate, alanine, triacylglycerol, and ceramides in oocytes and follicular cells of mares of different ages. Among them, glutamate is closely related to glutathione synthesis, redox homeostasis, and mitochondrial function; alanine can reflect glycolysis and anaerobic metabolic activity, while changes in triacylglycerol and ceramides suggest the remodeling of lipid reserve utilization and membrane lipid signal transduction status.

At present, there are many gaps in the research on the reproductive performance and estrus regulation of Kazakh horses. Therefore, this study aimed to characterize metabolic differences across stages of synchronized estrus in Kazakh mares and to identify candidate metabolic markers and pathways associated with follicular development, providing technical support and theoretical references for optimizing the handling plan of synchronous estrus in Kazakh horses, improving the conception rate during mating, and promoting the large-scale development of the horse industry.

Estrus synchronization protocols have been widely optimized and routinely applied in cattle and sheep breeding systems, with standardized schemes such as Ovsynch achieving stable reproductive efficiency in dairy cows, and melatonin + progestagen vaginal implants effectively inducing estrus in anestrous ewes out of breeding season [15,16]. However, estrus synchronization technology for mares remains far less standardized and popularized compared with ruminants, limited by unique equine estrous cycle characteristics, inconsistent follicular development response to exogenous hormones, and insufficient basic metabolic research supporting protocol optimization. For local Kazakh horses in Xinjiang, unstable estrus synchronization effects and low conception rates severely restrict large-scale intensive breeding, highlighting the urgent need to uncover serum metabolic regulatory patterns across successive synchronization stages.

The present study therefore set three specific research aims: (1) to profile the dynamic differences in serum metabolome of Kazakh mares at four successive key stages of estrus synchronization (M1, M3, P, L); (2) to screen stage-specific significantly differential metabolites (DAMs) and identify hub endogenous metabolites closely correlated with reproductive steroid hormones; (3) and to clarify the core metabolic pathways dominating follicular development during estrus synchronization, and provide theoretical support for optimizing estrus synchronization schemes and stage-targeted feeding management of Kazakh horses. Untargeted metabolomics technology was applied to complete the above research objectives.

## 2. Materials and Methods

### 2.1. Experimental Design and Sample Collection

#### 2.1.1. Animal Selection, Inclusion/Exclusion Criteria, Housing and Feeding Management

This experiment was carried out during the natural equine breeding season (May–August 2025, Tacheng, Xinjiang, China). The local natural photoperiod throughout the trial was approximately 15 h light:9 h dark, which supports physiological follicular development and regular cyclicity in mares. Six healthy adult Kazakh mares aged 4–7 years with a body weight of 380 ± 50 kg were selected for longitudinal repeated sampling. Strict inclusion and exclusion criteria were applied to standardize experimental subjects, as follows.

Inclusion criteria: Regular spontaneous estrous cycles confirmed by serial transrectal ultrasonography prior to the trial; normal ovarian and uterine morphology; consistent body condition score (BCS) of 3.0–3.5/5; pluriparous with no recent foaling within 6 months; and free of clinical lameness or chronic metabolic disorders.

Exclusion criteria: Mares with reproductive tract inflammation, ovarian cysts, historical dystocia, severe parasitic infection, or any systemic drug/hormone administration within the preceding 3 months; lactating mares and seasonally anestrous individuals were excluded.

All mares were maintained under unified housing and feeding conditions throughout the whole trial period. Animals were housed in open barns with free access to outdoor paddocks during daytime; clean drinking water was available ad libitum. The daily diet consisted of local forage roughage supplemented with commercial concentrated feed (crude protein 14.0%) fed twice daily at fixed times. Routine deworming and equine core vaccination schedules were implemented one month before the experiment commenced. No dietary adjustments or additional nutritional supplements were introduced during estrus synchronization treatment to eliminate metabolic confounding factors.

#### 2.1.2. Stepwise Estrus Synchronization Protocol with Full Reagent Specifications

A standardized 14-day progesterone intravaginal device + PGF_2_α + hCG synchronization protocol was implemented sequentially from Day 0 (device insertion) to tertiary follicle emergence, with all intervention timings, doses, administration routes, product commercial names, manufacturers, and technical specifications fully documented below.

Day 0 (M1 sampling time point): A progesterone-releasing intravaginal device containing 1.96 g of progesterone (commercial name: Progesterone Injection, manufacturer: NSHF, Ningbo, China) was inserted into the vaginal tract of each mare to suppress endogenous follicular activity and synchronize luteal status. Jugular venous blood sampling was performed immediately after device insertion to generate the M1 group dataset.

Day 14 (M3 sampling time point): The progesterone intravaginal device was completely removed. Immediately after device withdrawal, each mare received an intramuscular injection of 0.3 mg prostaglandin F_2_α (PGF_2_α; product name: Cloprostenol Sodlum Injection, manufacturer: NSHF, China). Whole blood was collected to establish the M3 sample group on the same day of device removal and PG injection.

24 h post-PGF_2_α injection (P sampling time point): Human chorionic gonadotropin (hCG; product name: Chorionic Gonadotrophin For Injection, manufacturer: NSHF, China) was administered via intramuscular injection to induce coordinated ovulatory signaling, consistent with the published synchronization workflow. Blood samples were collected 24 h after PG injection to form the P group.

All hormone injections were performed by a licensed equine veterinarian using standardized intramuscular injection sites on the neck musculature. No off-label dosage or alternative administration routes were applied to any experimental subject.

#### 2.1.3. Independent Paragraph: Transrectal Reproductive Ultrasonography Monitoring

Transrectal ultrasonographic reproductive examinations were conducted as a separate standardized monitoring procedure throughout the synchronization trial to track follicular dynamics and define the final L sampling time point.

Ultrasonography was performed using a veterinary ultrasound scanner (Model American imported veterinary B-ultrasound Ibex Pro, Carlsbad, CA, USA) equipped with a 7.5 MHz linear rectal probe. Examination schedules were fixed as follows: biweekly scanning from Day 0 to Day 13 of progesterone device retention; daily transrectal ultrasound monitoring starting on Day 14 (device removal) until tertiary follicle detection.

Reproductive parameters recorded during each scan included ovarian volume, corpus luteum presence/diameter, number and diameter of all antral follicles, and endometrial thickness. A follicle with a diameter ≥ 30 mm was defined as a tertiary follicle in the present study; the moment when the first tertiary follicle was clearly identified via ultrasound was designated as the L sampling stage. Clinical behavioral estrus evaluation (teasing with a stallion) was also recorded alongside each ultrasound assessment to cross-validate ovarian activity.

#### 2.1.4. Group Allocation Design and Blood Sample Collection Procedures

The present study adopted a longitudinal repeated-measures experimental design rather than random allocation of independent animals to discrete treatment groups. The identical six Kazakh mares were sequentially sampled at four predefined physiological stages (M1, M3, P, L) determined by the unified hormone intervention timeline and real-time ultrasonographic follicle monitoring; no randomization between individuals was required, as all time-series samples originated from the same cohort of experimental subjects.

At each of the four sampling time points (M1, M3, P, L), approximately 10 mL of whole blood was collected from the jugular vein of each mare using heparin sodium anticoagulant vacuum tubes. Blood samples were gently inverted 5–8 times for uniform anticoagulant mixing and immediately centrifuged at 3000 r/min for 10 min at room temperature. The separated upper serum layer was aliquoted into 1.8 mL cryogenic vials, snap-frozen in liquid nitrogen, and subsequently transferred to a −80 °C ultra-low temperature freezer for long-term storage until untargeted metabolomic profiling analysis. All serum separation and freezing operations were completed within 30 min post-blood collection to prevent metabolite degradation.

### 2.2. Metabolite Extraction and Sequencing

The plasma samples were separated using a Vanquish ultra-high-performance liquid chromatography (UHPLC) system equipped with a Hypersil GOLD C18 column (Thermo Fisher Scientific, Waltham, MA, USA). The chromatographic separation was carried out under the conditions of a column temperature of 40 °C and a flow rate of 0.2 mL/min. In the positive ion mode, the mobile phase A was an aqueous solution containing 0.1% formic acid, and the mobile phase B was methanol; in the negative ion mode, the mobile phase A was a 5 mmol/L ammonium acetate aqueous solution, and the mobile phase B remained methanol. To comprehensively interpret the identified metabolites, multiple public databases were used for functional annotation, including the Kyoto Encyclopedia of Genes and Genomes (KEGG; https://www.genome.jp/kegg/pathway.html, accessed on 5 May 2026), the Human Metabolome Database (HMDB; https://hmdb.ca/metabolites, accessed on 5 May 2026), and LIPID Maps (http://www.lipidmaps.org/). Metabolite annotation was classified following Metabolomics Standards Initiative (MSI) criteria; all reported differential metabolites reached MSI Level 2 (putative identification via accurate mass, MS/MS fragmentation, and database matching).

### 2.3. Screening and Enrichment Analysis of Differential Metabolites

The metabolomics data were preprocessed using the metaX software (v1.4.1, [17]), followed by multivariate statistical analysis. Unsupervised mode: Principal Component Analysis (PCA) was used to evaluate the natural clustering trend among sample groups; Supervised mode: Partial Least Squares Discriminant Analysis (OPLS-DA) was used to calculate the importance of variable projections (Variable Importance in Projection, VIP). Metabolites with significant differences (|log_2_Fold change| ≥ 0.5, VIP ≥ 1 and *P*adj < 0.05) were subjected to KEGG pathway enrichment analysis through MetaboAnalyst (https://www.metaboanalyst.ca/). The thresholds |log_2_FC| ≥ 0.5, VIP ≥ 1 and *P*adj < 0.05 were selected according to widely accepted standards in equine metabolomics research to balance screening stringency and retention of biologically relevant metabolites [18].

### 2.4. DAMs Mfuzz Timing Analysis

The metabolite data were sorted in Excel and then imported into the R environment (version 4.5.2). The mean values of each metabolite within the same group were calculated. Subsequently, the mean value matrix was standardized using Z-score to make the mean expression level of each metabolite across different samples 0 and the standard deviation 1, thereby eliminating the influence of the scale. Replace zero imputation with k-nearest neighbor (k-NN) missing value imputation to reduce clustering bias. Then, the fuzzy C-means clustering (Mfuzz) was used to perform trend clustering on the standardized metabolite expression matrix. The number of clusters (k) was determined by integrating multiple methods: calculate the first-order difference of the within-group sum of squares (WSS) for k = 2 to 10, and take the k corresponding to the minimum difference; calculate the average silhouette width for each k value, and take the k corresponding to the maximum value; calculate the within-group error sum of squares for each k value, and take the k corresponding to the minimum value. Based on the results of these three methods, the final number of clusters was determined by the majority voting principle. The fuzzy parameter m of Mfuzz was estimated using the mestimate function. Each metabolite was assigned to the cluster with the highest membership degree, and the membership degree matrix of each cluster was output.

### 2.5. Hub DAMs Screening

The Pearson correlation coefficients between the expression trends of each metabolite and the cluster center curves were calculated using RStudio (R-4.5.2). The metabolites with the absolute values of the correlation coefficients ranking in the top 10% within each cluster were selected as the core metabolites of that cluster. The top 10% correlation threshold was applied to retain metabolites with the strongest temporal expression correlation to cluster trends, consistent with previous Mfuzz hub metabolite screening pipelines in animal reproductive metabolomics [19]. The Hub DAMs were subjected to enrichment analysis. The metabolite names were batch-mapped to the KEGG database (Kyoto Encyclopedia of Genes and Genomes). The enrichment analysis employed the hypergeometric test, with *p*-values corrected using the Benjamini–Hochberg method. A *P*adj value less than 0.05 after correction was considered as significant enrichment. Each cluster separately outputs the KEGG pathways that were significantly enriched.

## 3. Results and Analysis

### 3.1. Screening of Differentially Expressed Metabolites in Stages M1 to M3

**Figure 1:**
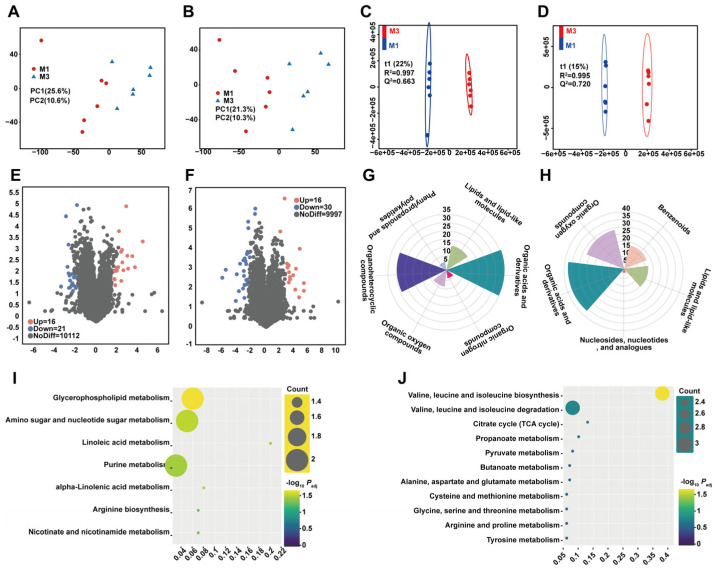
M1 to M3 Screening and enrichment analysis of differential metabolites. (**A**,**B**) PCA positive and negative ion plots; (**C**,**D**) positive and negative ion OPLS-DA; (**E**,**F**) volcano plot for metabolite screening; (**G**,**H**) metabolite classification radar plot; (**I**,**J**) KEGG pathway enrichment analysis of differential metabolites.

The differences among the various sample groups were evaluated using PCA and OPLS-DA. The permutation test (n = 200 permutations) confirmed no overfitting; the intercept of the Q^2^ regression line was below zero for both ion modes, validating model reliability alongside R^2^ and Q^2^ values. The PCA results showed that the blood samples of Kazakh horses in both positive and negative ion patterns exhibited intra-group clustering and significant separation between groups (positive ions: PC1 = 25.6%, PC2 = 10.6%; negative ions: PC1 = 21.3%, PC2 = 10.3%), indicating significant differences in plasma metabolites of Kazakh horses at different stages (Figure 1A,B). The model was further tested through an OPLS-DA permutation test. The stability parameters of the M1 vs. M3 model were positive ions R^2^ = 0.997 and negative ions R^2^ = 0.995, and the prediction ability parameters were positive ions Q^2^ = 0.663 and negative ions Q^2^ = 0.720, indicating a good fit of the model and that the metabolomics data can be used for further analysis (Figure 1C,D). VIP > 1 and *P*adj < 0.05 were used as the criteria for screening differentially expressed metabolites. In the positive ion pattern, 37 significantly differentially expressed metabolites were identified between M1 and M3, with 16 significantly upregulated and 21 significantly downregulated (Figure 1E,F). In the negative ion pattern, 47 significantly differentially expressed metabolites were identified between M1 and M3, with 17 significantly upregulated and 30 significantly downregulated. The chemical classification radar plot of differentially expressed metabolites showed that the differentially expressed metabolites were mainly lipids and lipid molecules, organic acids and their derivatives, as well as organic oxides, organic heterocyclic compounds, benzene compounds, and nucleosides, etc. (Figure 1H) (see Supplementary Material Table S1).

KEGG pathway enrichment analysis was performed on the differential metabolites in the M1 vs. M3 stage. The results showed that the positive ion mode metabolites were mainly enriched in glycerophospholipid metabolism, amino sugar and nucleoside sugar metabolism, linoleic acid metabolism, purine metabolism, α-linolenic acid metabolism, arginine biosynthesis, nicotinic acid and nicotinamide metabolism, etc. (*P*adj < 0.05). The negative ion mode metabolites were mainly significantly enriched in amino acid metabolism (biosynthesis of valine, leucine and isoleucine, degradation of valine, leucine and isoleucine, metabolism of alanine, aspartate and glutamate, cysteine and methionine metabolism, metabolism of glycine, serine and threonine, arginine and proline metabolism, tyrosine metabolism, *P*adj < 0.05); energy metabolism (tricarboxylic acid cycle, propionic acid metabolism, pyruvic acid metabolism, butyric acid metabolism, *P*adj < 0.05) (Figure 1I,J) (see Supplementary Material Table S2).

### 3.2. Screening of Differentially Metabolized Substances from the M3 to P Stage

**Figure 2:**
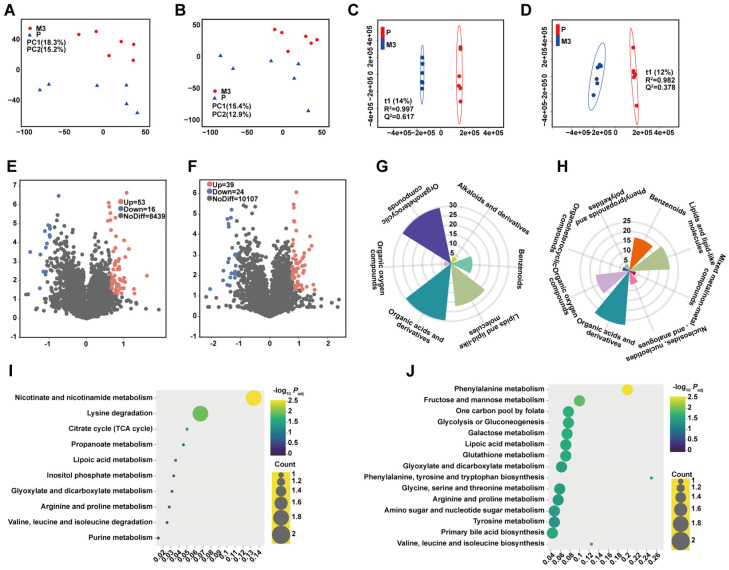
M3 to P Screening and enrichment analysis of differential metabolites. (**A**,**B**) PCA positive and negative ion plots; (**C**,**D**) positive and negative ion OPLS-DA; (**E**,**F**) volcano plot for metabolite screening; (**G**,**H**) metabolite classification radar plot; (**I**,**J**) KEGG pathway enrichment analysis of differential metabolites.

The differences among the various sample groups were evaluated through PCA and OPLS-DA analysis. The results showed that the plasma samples of Kazakh horses in both positive and negative ion modes exhibited intra-group clustering and significant separation between groups (positive ions: PC1 = 18.3%, PC2 = 15.2%; negative ions: PC1 = 15.4%, PC2 = 12.9%), indicating significant differences in metabolites at different stages of Kazakh horses (Figure 2A,B). The model was further tested through OPLS-DA permutation test, with the stability parameters of the M3 vs. P model being positive ions R^2^ = 0.997, negative ions R^2^ = 0.982, and the prediction ability parameters being positive ions Q^2^ = 0.617, negative ions Q^2^ = 0.378. Although the negative ion mode Q^2^ value was relatively moderate (0.378), permutation testing excluded overfitting, and the model still separated M3 and P samples clearly for exploratory metabolomic comparison. The metabolomics data could be used for further analysis (Figure 2C,D). Based on the results of OPLS-DA, VIP > 1 and *P*adj < 0.05 were used as the criteria for screening differential metabolites. The results showed that M3 vs. P positive ions identified 69 significantly different metabolites, 53 of which were significantly upregulated, and 16 were significantly downregulated (Figure 2E,F). M3 vs. P negative ions identified 63 significantly different metabolites, 39 of which were significantly upregulated, and 24 were significantly downregulated. The chemical classification radar plot of differential metabolites showed that the differential metabolites at the M3 and P stages were mainly distributed in three categories: organic heterocyclic compounds, organic acids and their derivatives, and lipid and lipid-like molecules. Among them, organic heterocyclic compounds and organic acids and their derivatives accounted for the highest proportion, followed by lipid classes (Figure 2G,H).

KEGG pathway enrichment analysis was performed on the differential metabolites in the M3 vs. P stage (Figure 2I,J). The results showed that the positive ion mode metabolites were mainly enriched in pathways such as nicotinate and nicotinamide metabolism, lysine degradation, citric acid cycle (tricarboxylic acid cycle, TCA cycle), propionic acid metabolism, lipoic acid metabolism, inositol phosphate metabolism, acetaldehyde and dicarboxylic acid metabolism, arginine and proline metabolism, valine, leucine and isoleucine degradation, purine metabolism, etc. (*P*adj < 0.05). The negative ion mode metabolites were mainly significantly enriched in pathways such as phenylalanine metabolism, fructose and mannose metabolism, folate-mediated one-carbon unit metabolism, glycolysis or gluconeogenesis, galactose metabolism, lipoic acid metabolism, glutathione metabolism, acetaldehyde and dicarboxylic acid metabolism, biosynthesis of phenylalanine, tyrosine and tryptophan, glycine, serine and threonine metabolism, arginine and proline metabolism, amino sugar and nucleotide sugar metabolism, tyrosine metabolism, primary bile acid biosynthesis, valine, leucine and isoleucine biosynthesis, etc. (*P*adj < 0.05).

### 3.3. Screening of Differential Metabolites in Phases P to L

**Figure 3:**
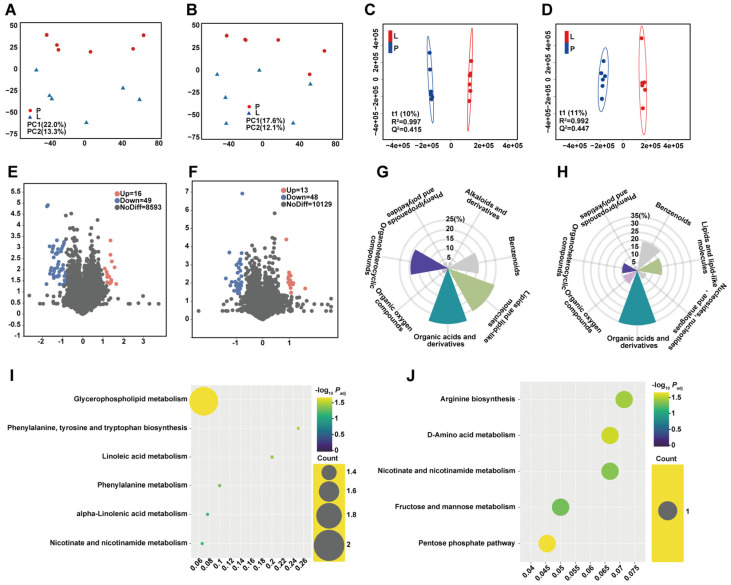
P to L Screening and enrichment analysis of differential metabolites. (**A**,**B**) PCA positive and negative ion plots; (**C**,**D**) positive and negative ion OPLS-DA; (**E**,**F**) volcano plot for metabolite screening; (**G**,**H**) metabolite classification radar plot; (**I**,**J**) KEGG pathway enrichment analysis of differential metabolites.

The differences among the various sample groups were evaluated using PCA and OPLS-DA. The PCA results showed that the blood samples of Kazakh horses in both positive and negative ion patterns exhibited intra-group clustering and significant separation between groups (positive ions: PC1 = 22.0%, PC2 = 13.3%; negative ions: PC1 = 17.6%, PC2 = 12.1%), indicating significant differences in plasma metabolites of Kazakh horses at different stages (Figure 3A,B). The model was further tested through an OPLS-DA permutation test. The stability parameters of the P vs. L model were positive ions R^2^ = 0.997, negative ions R^2^ = 0.992, and the prediction ability parameters were positive ions Q^2^ = 0.415, negative ions Q^2^ = 0.447. This indicates that the model was well established and could accurately reflect the objective facts. Metabolomics data can be used for further analysis (Figure 3C,D). Based on the results of OPLS-DA, the variable projection importance VIP > 1 and *P*adj < 0.05 were used as the criteria for screening differential metabolites. The results (Figure 3E,F) showed that 65 significantly different metabolites were identified in the P vs. L positive ions, 16 significantly upregulated, and 49 significantly downregulated. Overall, 61 significantly different metabolites were identified in the P vs. L negative ions, 13 significantly upregulated and 48 significantly downregulated.

The radar chart for the chemical classification of differential metabolites (Figure 3G,H) shows that the differential metabolites between P and L are mainly centered around organic acids and their derivatives, with lipids and lipid-like molecules, organic heterocyclic compounds, and benzene compounds as important components. This indicates that the metabolic changes at this stage are centered around the activation of energy metabolism, accompanied by the coordinated remodeling of lipid metabolism and aromatic amino acid metabolism, providing sufficient material and energy support for the rapid growth and maturation of the tertiary follicles. KEGG pathway enrichment analysis of the differential metabolites at the P vs. L stage (Figure 3I,J) shows that positive ion mode metabolites are mainly enriched in glycerophospholipid metabolism, phenylalanine, tyrosine and tryptophan biosynthesis, linoleic acid metabolism, phenylalanine metabolism, α-linolenic acid metabolism, nicotinate and nicotinamide metabolism, etc. (*P*adj < 0.05). Negative ion mode metabolites are mainly significantly enriched in arginine biosynthesis, D-amino acid metabolism, nicotinate and nicotinamide metabolism, fructose and mannose metabolism, and the pentose phosphate pathway, etc. (*P*adj < 0.05).

### 3.4. Time Series Analysis of DAMs and Selection of Hub DAMs

**Figure 4:**
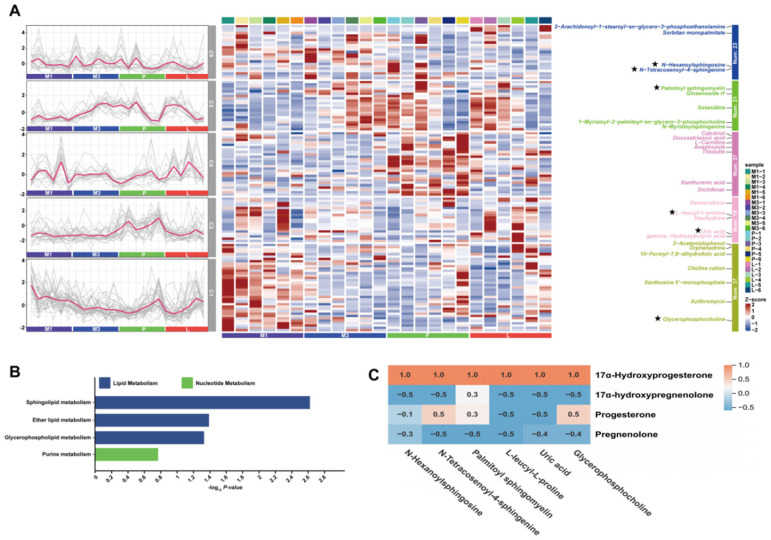
(**A**) illustrates the dynamic changes in differential metabolites (DAMs) during the four stages of M1, M3, P, and L. (**B**) shows the results after KEGG pathway enrichment of these temporal change metabolites. The abscissa represents −log10(*p* value), with a larger value indicating a more significant enrichment of the pathway; the ordinate represents the names of the enriched metabolic pathways. (**C**) is a correlation heatmap used to display the relationships between the selected Hub DAMs and several hormone indicators. (★) are the candidate key differential metabolites (Hub DAMs) obtained through subsequent screening.

To reveal the dynamic change patterns of differential metabolites over time, this study conducted Mfuzz time-series clustering analysis on the differential metabolites expressed in M1, M3, P, and L. The results showed that the differential metabolites could be clearly clustered into 5 clusters with different expression trends (C1–C5), each containing 37, 19, 27, 21, and 23 metabolites (Figure 4A). The heatmap results further indicated that the relative abundance change trends of metabolites in different clusters varied significantly at each stage sample, suggesting that these differential metabolites may play different regulatory roles during estrus synchronization and follicular development. The representative metabolites in each clustering cluster are listed on the right side of Figure 4A, such as N-hexanoylsphingosine, L-leucyl-L-proline, and uric acid, which visually demonstrate the unique time-series change patterns of each cluster. The metabolites marked with stars (★) are the candidate key differential metabolites (Hub DAMs) obtained through subsequent screening. The time-series clustering of the differential metabolites obtained from the comparisons of M1 vs. M3, M3 vs. P, and P vs. L showed that they were clustered into 5 clusters (C1–C5), each containing 37, 19, 27, 21, and 23 metabolites. The Hub metabolites include N-Hexanoylsphingosine, N-Tetracosenoyl-4-sphingenine, Palmitoyl sphingomyelin, L-leucyl-L-proline, Uric acid, and Glycerophosphocholine, etc.

The results of KEGG pathway enrichment analysis (Figure 4B) indicate that these time-series differential metabolites are mainly enriched in pathways such as sphingolipid metabolism, ether lipid metabolism, glycerophospholipid metabolism, and purine metabolism, among which the lipid metabolism-related pathways are the most abundant. Further correlation analysis of the selected Hub DAMs and hormone indicators was performed (Figure 4C). Pearson correlation coefficients between six authentic endogenous hub metabolites and four reproductive steroid hormones were calculated and summarized in Table S3. 17α-hydroxyprogesterone exhibited strong positive correlations (r = 1.00, *P*adj < 0.05) with all screened hub metabolites. N-tetracosenoyl-4-sphingenine and glycerophosphocholine showed moderate positive correlations with progesterone (r = 0.50, *P*adj < 0.05), while N-hexanoylsphingosine presented a weak positive correlation with progesterone (r = −0.10, *P*adj > 0.05). All hub metabolites displayed negative correlations with pregnenolone and 17α-hydroxypregnenolone, with correlation coefficients ranging from −0.50 to −0.30. Full quantitative r values and adjusted *p*-values for each metabolite-hormone pair are provided in Table S4 for transparent data presentation.

Correlation coefficient r range: −1~1; r > 0 = positive correlation, r < 0 = negative correlation; |r| closer to 1 means stronger correlation.

Significance test: All correlation analyses used Benjamini–Hochberg method to calculate adjusted *p*-values (*P*adj).

## 4. Discussion

The results of this study show that there are clear differences in the metabolism of Kazakh horses among the M1, M3, PG, and tertiary follicle stages. Among them, the M1 vs. M3 stage shows the largest difference in the number of metabolites, indicating that the period of hormone implantation maintenance is the most significant period for the body’s metabolic adjustment. From the M3 to the PG stage, the main focus is on increasing metabolites, indicating that after progesterone device removal and PGF2α treatment, the body transitions from an inhibited state to a restored state. From the PG stage to the tertiary follicle stage, the main manifestation is an increase in lipid, amino acid, and energy metabolism, indicating that the body has entered a metabolic state that supports the continued development of the follicle. This is consistent with the research results showing significant fluctuations in the fluid metabolic group of mares under different reproductive conditions [20,21].

The differences in serum metabolic profiles between the M1 and M3 stages were the most significant. A total of 129 and 120 different metabolites were identified in the positive and negative ion modes, respectively, and the down-regulated metabolites were predominant. KEGG enrichment analysis showed that the differentially expressed metabolites in this stage mainly involved glycerophospholipid metabolism, amino sugar and nucleotide sugar metabolism, linoleic acid metabolism, purine metabolism, branched-chain amino acid metabolism, tricarboxylic acid cycle, and pyruvate metabolism, etc. Glycerophospholipid and fatty acid metabolism are closely related to cell membrane renewal, signal transduction, and the supply of substrates for steroid hormone synthesis. The tricarboxylic acid cycle and pyruvate metabolism directly affect the body’s energy supply [22,23]. Therefore, the significant changes in a large number of lipid and energy metabolites from the M1 to M3 stages suggest that the mare in the embolus retention period is in a relatively obvious metabolic inhibition state. Compared with M1 vs. M3, the number of differentially expressed metabolites in M3 vs. PG decreased to 81 positive ions and 75 negative ions, but the majority were up-regulated metabolites, indicating that after embolus removal and administration of PG, the mare’s body began to transition from the previous inhibition state to the recovery state. The differentially expressed metabolites in this stage mainly concentrated on organic heterocyclic compounds, organic acids and their derivatives, and lipid and lipoid molecules. The enriched pathways mainly included nicotinic acid and nicotinamide metabolism, tricarboxylic acid cycle, phenylalanine metabolism, and glycolysis/glycogenolysis. Nicotinic acid and nicotinamide metabolism are involved in the generation of NAD^+^/NADPH, and NAD^+^/NADPH is closely related to mitochondrial energy supply, redox homeostasis, and steroid hormone synthesis. Therefore, the enrichment of related pathways in this stage suggests that after embolus removal, the mare’s body has begun to restore energy and reduce the power supply related to follicle development [24,25].

During the PG to tertiary follicle stage, 77 and 64 differential metabolites were identified under the positive and negative ion modes, respectively. The differential metabolites mainly consist of organic acids and their derivatives, accompanied by concurrent changes in lipids, lipoid molecules, organic heterocyclic compounds, and benzene compounds. KEGG enrichment analysis revealed that this stage was mainly enriched in pathways such as glycerophospholipid metabolism, arginine biosynthesis, phenylalanine metabolism, and carbohydrate metabolism. This indicates that the metabolic focus of the organism has shifted from “restoration” to “supply”, providing more sufficient structural substrates and energy support for the rapid formation and continuous development of the tertiary follicle. Li et al. [26] conducted a study on the follicular fluid of Mongolian horses and found that during the follicular development process, the protein and metabolic profiles were continuously reshaped, with particularly significant changes in lipid and amino acid metabolism. Abdoon et al. [27] also conducted a study on the follicular fluid of the single-humped camel and discovered that the metabolic differences between the breeding season and the non-breeding season mainly concentrated in lipid metabolism, energy metabolism, and the related networks of the follicular microenvironment, further indicating that the transition of reproductive status often accompanies significant metabolic reprogramming. Qiao et al. [28] conducted a metabolic profile study on the concurrent estrus of Guibei Ma sheep and found that the differential metabolites after treatment mainly involved energy metabolism and steroid synthesis. Wang et al. [29] also pointed out that after the withdrawal of progesterone, the hormone secretion and metabolic state in the animal would undergo redistribution. Hojo et al. [30] conducted a study on Japanese black cattle and also suggested that the timing of CIDR treatment and withdrawal would affect the estrus interval and the risk of silent estrus. Mehallaine et al. [31] conducted a study on Oulade Jerald ewes and found that different hormone combinations would affect the estrus initiation time and reproductive outcome. Although the species were different, this still indicated that the transition from the progesterone-inhibited state to the estrus recovery state was a key link in the concurrent estrus program. At the same time, Rodríguez et al. [32] conducted a study on beef cattle and found that during the implementation of the progesterone, estradiol, and eCG synchronous program, some metabolic indicators such as blood glucose and NEFA would also change, even though the final reproductive outcome may not be synchronized, it also suggested that the synchronous program itself could cause the adjustment of the body’s metabolic state.

The differential metabolites exhibited distinct dynamic change patterns in the M1, M3, PG, and tertiary follicle stages, and were classified into 5 categories. Representative metabolites include N-hexanoyl sphingosine, N-24-carotenoyl-4-sphingosine, palmitoyl sphingomyelin, L-leucyl-L-proline, uric acid, and glycerophosphocholine, etc. The KEGG enrichment analysis indicated that these temporal differential metabolites were mainly enriched in sphingolipid metabolism, lipid metabolism, glycerophospholipid metabolism, and purine metabolism, among which the lipid metabolism-related pathways were the most significant. This suggests that membrane lipid remodeling and purine metabolism were involved throughout the entire process of estrus synchronization. Previous studies have shown that sphingolipids and glycerophospholipids not only affect the fluidity of cell membranes and signal transduction, but are also closely related to steroidogenesis, mitochondrial function, and the developmental potential of oocytes [33,34,35]. Further correlation analysis revealed that 17α-hydroxyprogesterone was positively correlated with most Hub DAMs, while 17α-hydroxypregnenolone was negatively correlated with most Hub DAMs; moreover, progesterone was positively correlated with some sphingolipid metabolites. These coordinated variations mirror synchronized shifts between steroid precursor concentrations and lipid metabolic profiles during estrus synchronization, rather than confirming direct causal regulatory effects. In particular, uric acid, a significantly altered metabolite identified as a hub DAM, co-varies with purine catabolism status and may participate in the modulation of redox balance and metabolic stress across consecutive estrus stages. De Souza Oliveira et al. [36] demonstrated that circulating gonadal steroid concentrations dynamically shift alongside ovarian follicle maturation; the observed hub DAM fluctuations are therefore proposed as key metabolic markers co-associated with endocrine remodeling and follicular developmental progression, rather than direct upstream drivers of follicle growth.

During the M1 to M3 stages, the main manifestations are an increase in progesterone and a decrease in FSH/LH. Biochemically, there is an increase in bilirubin and uric acid while protein and some energy metabolism indicators decrease. The metabolic profile is mainly focused on lipid metabolism, purine metabolism, and energy metabolism remodeling, reflecting the inhibitory state under the influence of exogenous progesterone. From M3 to the PG stage, there is a decrease in progesterone and an increase in 17α-hydroxyprogesterone. Biochemical indicators gradually recover, and the metabolic profile is enriched in nicotinic acid and nicotinamide metabolism, the tricarboxylic acid cycle, and glycolipid metabolism, reflecting the recovery process after luteal degeneration. From PG to the tertiary follicle stage, there is an increase in CHOL, TG, and BUN in the low-progesterone background, and the metabolic profile is enriched in glycerophospholipid metabolism, amino acid synthesis, and sugar-lipid metabolism, reflecting the high metabolic support of the body for the rapid development of the tertiary follicle. The simultaneous estrus of Kazakh horses is not a simple fluctuation of hormone levels, but a continuous physiological process based on endocrine transformation as the driving force, with lipid–amino acid energy metabolism as the basis for coordinated remodeling. This result is basically consistent with the existing research conclusions on the regulation of the estrus cycle of mares, the metabolic characteristics of follicular fluid, and the biochemical connections between blood and follicular fluid [37,38]. From a production application perspective, this suggests that the management focus at different stages of the simultaneous estrus program should be differentiated. During the maintenance period of the implant, attention should be paid to the metabolic burden and redox state of the body. After the removal of the implant and PG treatment, attention should be paid to the restoration of energy and amino acid substrates, while in the stage of tertiary follicle formation, it is necessary to ensure the supply of lipid and energy substrates, which may provide preliminary metabolic clues to guide staged feeding management optimization in future large-scale breeding trials.

This study has several inherent limitations that should be noted for balanced interpretation of the findings and guidance for future research. First, the sample size of experimental Kazakh mares (n = 6) was relatively small, which may restrict the generalizability of the metabolite correlation results; larger cohort trials are required to verify the stability of screened hub markers. Second, only peripheral serum metabolomics was analyzed in this work, without simultaneous detection of follicular fluid or ovarian tissue metabolome, so we cannot directly confirm the local metabolic changes within ovarian follicles. Third, this study adopted untargeted metabolomics for differential metabolite screening, while absolute quantitative targeted metabolomics is needed to validate the expression abundance of hub DAMs in subsequent research. Fourth, all sampling was conducted during the natural breeding season of horses; further trials are required to explore metabolic regulatory patterns during seasonal anestrus. Fifth, the trial only focused on Kazakh horses, and whether these core metabolic pathways are conserved across other horse breeds remains unclear. Finally, this study only revealed correlative relationships between metabolites and reproductive hormones, and in vitro granulosa cell culture or in vivo metabolic intervention experiments are needed to verify causal regulatory mechanisms of key metabolic pathways.

## 5. Conclusions

This study fulfilled all three predefined research aims proposed in the Introduction via untargeted serum metabolomics analysis of Kazakh mares during estrus synchronization.

(1) Corresponding to Aim 1: We systematically characterized serum metabolome variations across M1, M3, P, and L stages; confirmed that lipids, organic acids, and heterocyclic compounds dominated stage-specific differential metabolites; and clarified the shifting metabolic characteristics from progesterone inhibition and luteolysis to tertiary follicle development.
(2) Corresponding to Aim 2: After filtering false-positive drug-derived metabolites, we screened a series of endogenous hub DAMs, including N-hexanoylsphingosine, palmitoyl sphingomyelin, L-leucyl-L-proline, and uric acid; correlation analysis verified their tight linkage with reproductive steroid hormone levels, which can serve as candidate metabolic markers for follicular development.
(3) Corresponding to Aim 3: KEGG enrichment identified nicotinate and nicotinamide metabolism, glycerophospholipid metabolism, amino acid biosynthesis and TCA cycle as core regulatory pathways mediating follicular growth. These lipid–amino acid energy metabolic networks coordinate the continuous physiological transition of mares during estrus synchronization.

In summary, this work systematically reveals the dynamic metabolic regulatory patterns during estrus synchronization in Kazakh horses, fills the breed-specific metabolomics research gap, and provides an important theoretical basis and practical reference for optimizing estrus synchronization protocols, adjusting stage-specific feeding management and improving conception rates of Kazakh horses under intensive breeding.

## Data Availability

The original contributions presented in this study are included in the article/Supplementary Materials. Further inquiries can be directed to the corresponding author.

## Supplementary Materials

The following supporting information can be downloaded at: https://www.mdpi.com/article/10.3390/ani16142222/s1, Table S1: positive and negative ion OPLS-DA; Table S2: KEGG enrichment analysis; Table S3: Names of differential metabolites between groups; Table S4: Hub DAMs and several hormone indicators.
